# Walk and listen: A multidimensional study on the soundscape of a University District

**DOI:** 10.1371/journal.pone.0343065

**Published:** 2026-02-20

**Authors:** Ilaria Grecchi, Giorgia Guagliumi, Oscar Azzimonti, Igor Costarelli, Antonio Sibilia, Giovanni Brambilla, Fabio Angelini, Roberto Benocci, Giovanni Zambon, Valentina Zaffaroni-Caorsi

**Affiliations:** 1 Department of Environmental and Earth Sciences, University of Milano-Bicocca, Milan, Italy; 2 Department of Sociology and Social Research, University of Milano-Bicocca, Milan, Italy; 3 Department of International and Political Sciences, University of Genoa, Genoa, Italy; Politecnico di Torino, ITALY

## Abstract

Human perception of a surrounding environment comes from our senses. Among these, vision has been considered the most important but, nowadays, the hearing perception features are attracting even more the attention of researchers. This study, based on five soundwalks conducted in the university district of Milano-Bicocca, compared subjective emotional responses evoked by the soundscape with psychoacoustic parameters determined from binaural recordings. Furthermore, a focus group discussion conducted at the end of each soundwalk made it possible to explore participants’ in-depth perceptions and to collect their accounts of everyday life in the neighbourhood, their geographical backgrounds, and their habitual and preferred soundscapes. From the survey analysis, a consistent preference emerged for green areas, which were also statistically clustered based on psychoacoustic indices, as well as the squares and the two sites most exposed to traffic, indicating alignment between subjective responses and psychoacoustic structure. Moreover, sites with comparable A-weighted sound pressure levels (dBA) elicited different perceptual evaluations: environments featuring water sounds were systematically perceived as less noisy, while the sites with the highest dBA levels were perceived either as chaotic or monotonous, depending on the listener’s subjective interpretation and the perceived meaning of the dominant noise source. These results reinforce the hypothesis that sound perception is shaped by contextual and semantic factors, and cannot be fully captured by conventional acoustic metrics alone.

## Introduction

Urban spaces are the scene of a multiplicity of artificial, natural and anthropogenic sounds. The diversity of social groups and populations (residents, commuters, city users, ... ) makes the urban soundscape a controversial issue in contemporary cities. In addition to the distributional issues related to traffic noise [1], urban sounds and the ways in which they are produced and perceived can denote forms of exclusion and social differentiation, but also generate a sense of belonging [2,3]. Soundscape quality is connected to public health and overall quality of life through a combination of ecosystemic (e.g., biodiversity), psychological (e.g., individual emotional responses), social (e.g., sense of belonging and place-based social values), and economic (e.g., economic valuation of place, dynamics of gentrification) factors [4]. This complexity becomes particularly evident in urban areas where different social groups with various spatial practices and rhythms of life interact daily. In this sense, university neighbourhoods represent a dynamic context, where students have a strong influence on the local economy and social structure [5], serving as compelling case studies. In fact, university campuses serve as centers for knowledge creation, interaction and community building [6,7]. If well designed, university environments can encourage creative encounters in public spaces, including “quiet zones”, which are recognised for their role in rest, concentration, and psychophysical recovery, which facilitate both socialisation and education [8]. Numerous studies have shown that acoustic conditions significantly influence students’ listening skills [9], cognitive performance and mental well-being [10] making careful planning of sound spaces necessary.

However, the functional and spatial heterogeneity that characterises university areas makes soundscape analysis complex due to the wide range of uses, users and acoustic morphologies present [11]. Traditionally, the management of human exposure to noise has focused on the quantitative control of the physical characteristics of the acoustic environment. Interventions have been aimed at ensuring compliance with sound pressure limits set by regulations for daytime and night-time periods [12]. A measurable reduction in sound levels does not always equate to an actual improvement in users’ qualitative perception of space [13]. Added to this is the intrinsic variability in acoustic measurements, which can lead to differences in individual perceptions of noise in the same environment [14]. This highlights the importance of subjective perception in evaluating a place’s acoustic environment, considering sound not as a waste product, but as a resource to be used to improve people’s well-being and health [15].

As soundscape research has advanced, attention has gradually shifted towards a deeper understanding of the acoustic experience, emphasizing not only physical aspects, but also subjective and contextual ones. Since the early 2000s, this shift has promoted interdisciplinary synergies involving the natural and social sciences, as well as the humanities [16]. It has also led to large-scale international projects and the definition of the ISO standard on soundscapes [17], which recognises the importance of subjective perception and the context and meaning attributed to sounds. In this paradigm, humans are no longer considered passive receivers of sound, but active interpreters of their sound environment within complex spatial, social and temporal dynamics.

This study explores the interactions between place, psychoacoustic indicators and subjective experience, using the soundwalk methodology. The analysis focuses on the spatial and temporal variability of sounds and perceptions in a heterogeneous area of the University of Milano-Bicocca neighborhood (Italy), which includes university squares, busy intersections, public parks and commercial spaces. This article aims to understand how users perceive sound in different contexts of the university neighbourhood, in relation to psychoacoustic indices determined from binaural recordings, and how this relates to their daily practices and sound environments. Through a combination of in situ recordings, surveys, and focus group discussions, the article seeks to explore what students hear in the neighbourhood, how they perceive it, and the extent to which their perceptions are reflected in their daily routines and life experiences. This study provides key insights to better inform and support sustainable urban planning decisions.

## Materials and methods

### Study area

Bicocca is a former industrial district in the northern periphery of Milan, Italy. After a large-scale urban regeneration project started in the late 1990s, the district became one of the main university campuses in the city, hosting the newborn University of Milano-Bicocca. The core of the campus consists of seven buildings scattered across two main arterial roads, Viale dell’Innovazione and Viale Sarca, and the nearby railway station Greco-Pirelli. The surrounding area presents a functional mix of urban features, including both modern and historical residential buildings, commercial activities, cafés, restaurants, supermarkets, and bookstores. Public spaces such as squares, gardens, and parks contribute to the diversity and vibrancy of the soundscape.

### Soundwalk

For the purpose of this study, soundwalks were used as a structured perceptual method to explore the acoustic characteristics of the study area, combining standardized quantitative ratings (ISO 12913-2:2018) with contextual qualitative observations focused on participants’ experience of the acoustic environment. A total of five soundwalks were conducted across October and November 2024, each lasting approximately 80 minutes (2.2 km). To ensure an adequate temporal coverage of the campus soundscape, sessions were intentionally scheduled at different times of day. Two soundwalks were performed in October (21/10/2024 at 14:30 and 29/10/2024 at 10:00), covering respectively an afternoon and a morning period. Three additional soundwalks were carried out in November (21/11/2024 at 14:30; 22/11/2024 at 15:00; 28/11/2024 at 15:00). Afternoon sessions were selected in November to maintain thermal conditions comparable to the October afternoon survey, preventing potential discomfort during morning hours. This distribution allowed capturing both morning and early-afternoon acoustic conditions while balancing methodological needs with participants’ availability.

The study was approved by the Ethics Committee of the University of Milano-Bicocca, Italy. Participation in the soundwalks was entirely voluntary and based on informed consent. Written informed consent was obtained from all participants at the time of registration, through a dedicated enrollment form provided prior to participation. The enrollment form included an information notice on the processing of personal data in compliance with Article 13 of the EU General Data Protection Regulation (Regulation EU 2016/679).

**Fig 1:**
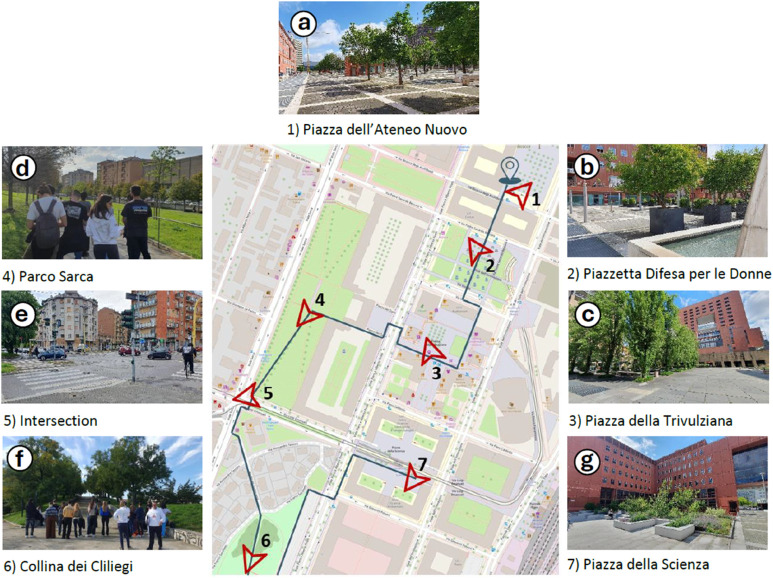
University District of Milano-Bicocca. The route is shown in blue, the stopping sites (1–7) in black, and the direction participants were asked to face during the recording and questionnaire completion is indicated in red.Basemap OpenStreetMap contributors (ODbL, https://www.openstreetmap.org/copyright). Map created with QGIS 3.38 Firenze.

The walks were carried out in small groups of approximately ten/fourteen participants (n=70 in total), mainly university students, who were considered particularly valuable due to their intuitive familiarity with the salient features of the local acoustic environment. This approach allows researchers, practitioners, policymakers and local authorities to collect and analyze perceptual and acoustic data grounded in the participants’ lived experience of the local sound environment [13]. In accordance with ISO 12913-2:2018 [17], each soundwalk was conducted by a trained moderator who guided the group along a predefined route (Fig 1) and provided clear instructions prior to the start of the activity. At specific points identified by the research team, participants were asked to stop and look in the same direction (Fig 1, red arrows), maintaining silence for two minutes and focusing on the surrounding soundscape. In accordance with the guidelines of the ISO 12913-2 standard, a minimum listening duration of three minutes is generally recommended. However, preliminary pilot tests revealed that participants had difficulty maintaining their attention for the entire three minutes and accurately recalling their perceptual impressions when completing the questionnaire immediately afterward.

During the two-minute listening sessions, a researcher equipped with SQope mobile binaural headphones (HEAD Acoustics), specifically designed for mobile acoustic measurements, collected binaural audio recordings (48 kHz and 24 bits). These headphones were connected to the researcher’s mobile device, where the HEAD B2U app was used to record and store the data. The audio files were then transferred to a computer and analyzed using the ArtemiS SUITE software platform. After each two-minute listening session, participants were invited to fill out an online questionnaire consisting of nine site-specific questions. During this phase, the moderator gave participants enough time to reflect and fill out the questionnaire without pressure, ensuring that realistic and spontaneous responses were collected. Once all participants had submitted their responses, the group moved on to the next stop. At the end of each soundwalk, a researcher conducted a short focus group with participants to further explore the subjective complexity of their experiences (see Section Focus group).

#### Spatial settings and questionnaire protocol.

The soundwalk route was carefully designed to include a variety of acoustic environments within the Bicocca campus, reflecting the heterogeneity of the area in terms of both soundscapes and spatial configurations. The selected locations differed in elevation (including sunken plazas, ground-level spaces, and elevated green areas) and acoustic features, representing the main functional, architectural, and social characteristics of the study area (Fig 1).

1. Piazza dell’Ateneo Nuovo (Fig 1a): a paved open square bordered by two university buildings and a construction site, with regularly planted trees
2. Piazzetta Difesa per le Donne (Fig 1b): a sunken plaza equipped with outdoor tables, featuring an artificial waterfall and an opening to the tram gallery below
3. Piazza della Trivulziana (Fig 1c): a sunken square lined with commercial establishments, integrating green areas and benches
4. Parco Sarca (Fig 1d): an urban park adjacent to a residential building and Viale Sarca, a major road
5. Intersection of Viale Sarca and Via Luigi Emanueli (Fig 1e): a road junction where multiple traffic lanes and a tram line converge
6. Collina dei Ciliegi (Fig 1f): an elevated green space within the park
7. Piazza della Scienza (Fig 1g): a car-free square enclosed by four university buildings (U1–U4) and intersected by tram tracks; it was recently subject to green redevelopment

Participants were asked to complete an Italian structured questionnaire immediately after each two-minute listening session. The translation of the eight soundscape perceptual attributes followed the validated Italian version proposed by [18] within the Soundscape Attributes Translation Project (SATP). In the first section, participants were asked to describe the audible sound sources (through an open-ended question) and to indicate the perceived predominance of these sources, as well as the presence or absence of sudden and short-duration acoustic events. Next, a series of questions using a five-point Likert scale were used to assess the perceived loudness of the environment, the degree to which the sound events were annoying or unpleasant, and how suitable the acoustic environment was for the location. The second section of the questionnaire focused on the perceptual evaluation of the soundscape based on attributes such as pleasantness, chaos, liveliness, stability, calmness, annoyance, dynamism and monotony. In addition, the final item asked whether participants would be willing to return to the location in the future (see SI1).

### Focus group

At the end of each soundwalk, participants were invited to take part in a focus group discussion held in a room inside university buildings. The meetings, moderated by a researcher, began with a general question about the experience of the walk and about the feelings it elicited. Few open questions were asked to delve into participants’ everyday practices in the district, their living environment and biographical experiences, and memories linked to sounds and noise (see S2). The focus group discussions lasted between 20 and 50 minutes and did not follow a predetermined structure, as the moderator tried not to interfere too much with the flow of the meetings. Since individual everyday practices and rhythms affect the ways people experience the city on foot [19], including the soundscape perception, the focus group methodology allowed us to explore complex aspects of the subjective dimension of soundscape that were difficult to include in the survey [20]. The focus group discussions were designed to examine students’ sound perceptions in depth, considering their different subjective features (e.g., geographical background, habitual soundscapes) and daily practices.

### Acoustic and psychoacoustics data processing

The acoustic data collection consisted of thirty-five 120 seconds (corresponding to 5 soundwalks with 7 stops each of 2 min) sound samples selected from the binaural sound recordings of real-life acoustic environments made during soundwalk experience in Bicocca university district. The acoustic data collected were extracted from binaural microphone’s corresponding software (Artemis of Head Acoustics). For each sound sample, a range of instantaneous psychoacoustic parameters were calculated according to the human auditory sensations following the ISO 12913-3:2019 [21] and [22], including Octave spectrum, Level (dBA) fast, loudness, roughness, fluctuation strength, sharpness and tonality. Except for the octave spectrum data, the left and right channel measurements were first averaged. Given the high similarity between the two, an additional averaging step was carried out. The considered indices were as follows:

- 1/3 Octave spectrum, a frequency analysis method that divides the audible range into bands, each spanning one-third of an octave, providing detailed information about the spectral content of a sound.
- dBA Level, physical measurement of sound pressure level which indicates the overall intensity or strength of the sound, reported in decibels (dB) as well as in A-weighted decibels (dB(A)) to account for the frequency sensitivity of human hearing.
- Loudness, which measures the auditory sensation on a scale from quiet to high intensity, taking into account the effects of auditory masking, spectral sensitivity, and nonlinearities, calculated according to DIN 45631/A1 standard [23].
- Roughness, related to fast modulation, which are quick temporal changes of sound DIN 38455 / ECMA-418-2.
- Fluctuation Strength, similar to roughness but related to slower modulations.
- Sharpness, a sensation of timbre related to the high frequency content, is calculated according to DIN 45631/A1 standard [23].
- Tonality, another sensation of timbre, indicating whether a sound consists of mainly tonal components, therefore equipped with a dominant frequency or broadband noise, calculated according to the Hearing Model ECMA-418-2.

### Acoustic and psychoacoustics data analysis

The binaural recordings obtained during the soundwalks were imported into the ArtemiS SUITE and analyzed by site and soundwalk. Descriptive and statistical analyses were conducted to explore the data. As the psychoacoustic data did not follow a normal distribution, nonparametric tests were employed to assess statistical significance between sites, including the Kruskal-Wallis test and post hoc Dunn’s test with p-value adjustment. Furthermore, A-weighted spectral levels in one-third octave bands were extracted across the entire frequency spectrum. The choice to extract A-weighted spectral levels was motivated by the fact that the study focuses on understanding human perception in the various sound environments encountered during the soundwalk, and therefore required a representation that accounts for the frequency sensitivity of human hearing. Considering that the recordings were binaural, both left and right channels were processed, and the energetic mean of the two channels was calculated to obtain a single representative spectrum per recording. To explore the spectral characteristics of each location, two types of visualizations were produced. First, to assess intra-site variability, all the 1/3 octave spectra corresponding to different soundwalk sessions were plotted together for each site. Then, to highlight inter-site differences, the mean spectrum was calculated for each measurement point by averaging the one-third octave band levels across all soundwalk sessions carried out at that specific site. This resulted in seven representative spectral curves, one per site, allowing for a direct comparison of the frequency content and overall acoustic profiles between different areas of the campus. To perform for the Clustering analysis, the dataset used included multiple observations of psychoacoustic indices per each of the 7 sites. A hierarchical agglomerative clustering (HAC) was performed using the Euclidean distance matrix. Using clValid package [24], we tested clustering solutions from 2 to 7, using different clustering algorithms: (i) “hierarchical”: hierarchical agglomerative clustering; (ii) “kmeans”: k-means clustering, (iii) “pam”: Partitioning Around Medoids, (iv) “model”: model-based clustering. A final validation measure was accessed using both “internal”, that evaluates clustering structure using the data itself, and “stability”, which assesses the robustness of the clustering. package. The choice of the number of clusters was primarily guided by the Silhouette index, which suggested 4 clusters as the optimal balance between cluster cohesion and separation. Finally, clusters were visualized using the factoextra package [25] to aid visual interpretation of the site segmentation.

### Survey data analysis

A database with soundwalks questionnaire and acoustic data was prepared, combining participants’ answers and psychoacoustic indicators from binaural registrations. The database contained 490 lines including the 70 respondents’ answers in each of the 7 sites. Descriptive analyses were carried out to depict the socio-demographic features of the sample. Frequencies, descriptive analyses and contingency tables were utilized to untangle the soundscape perception across the seven sites. The open questions on loudest noise sources and thoughts and feelings were analyzed with NVivo 15 word frequency tool. According to ISO/TS 12913-3:2019 (annex A-Method A) [21,26], affective responses have been represented using a two-dimensional model, where the horizontal axis is related to how pleasant or unpleasant the environment was judged (Pleasant), while the vertical axis is related to the amount of human and other activity (Eventful). The two additional axes of the circumplex model, rotated by 45° from the two main dimensions on the same plane, describe environments that are chaotic and stressful versus calm, and environments that are monotonous and dull versus vibrant and exciting [27,28].

### Combined acoustic/psychoacoustic and survey data

Spearman correlations were calculated to study the relationships between psychoacoustic indices and survey results, given the non normal distribution of the data. Statistical analyses were performed using SPSS (Version 29) and R.Studio Version (2025.05.1+513). Plots and graphic designs in this study were performed using R packages “dyplr”, “tidyr”, “tidyverse”, “ggpubr”, “ggplot2”, “rstatix”, “knitr” [25,29–33].

### Focus group data analysis

The focus groups contents were fully transcribed word-by-word, in order to preserve the expressive and semantic richness of the narratives that emerged. The analysis followed an inductive approach, which allows interpretative categories to emerge from the empirical material itself, rather than applying pre-existing theoretical frameworks [34]. This methodological orientation proved particularly useful for capturing subjective representations of urban spaces and the everyday practices that take place within them. Although a thematic framework was initially used - organized around three types of places: squares, parks, and intersections - the analysis followed a flexible and open logic, giving value to the meanings that participants attributed to the different spatial contexts. Within this framework, the dialogic interaction in the focus groups fostered the co-construction of collective narratives, making it possible to identify the distinctive features of each place, both in sensory and symbolic terms, which guided the design of the sound walk path.

## Results

### The respondents’ sample: sociodemographics and participants

The total number of participants was 70, out of which 30 declared themselves as women, 28 as men, 6 as non-binary and 6 preferred not to reply. All the respondents were familiar with the university and with the district: the vast majority were students or student workers (93%), while few respondents (7%) were PhD students. The sample’s mean age was 22, with a minimum of 18 and a maximum of 33 years old.

### Acoustic and psychoacoustic characterization of the different sites

**Fig 2:**
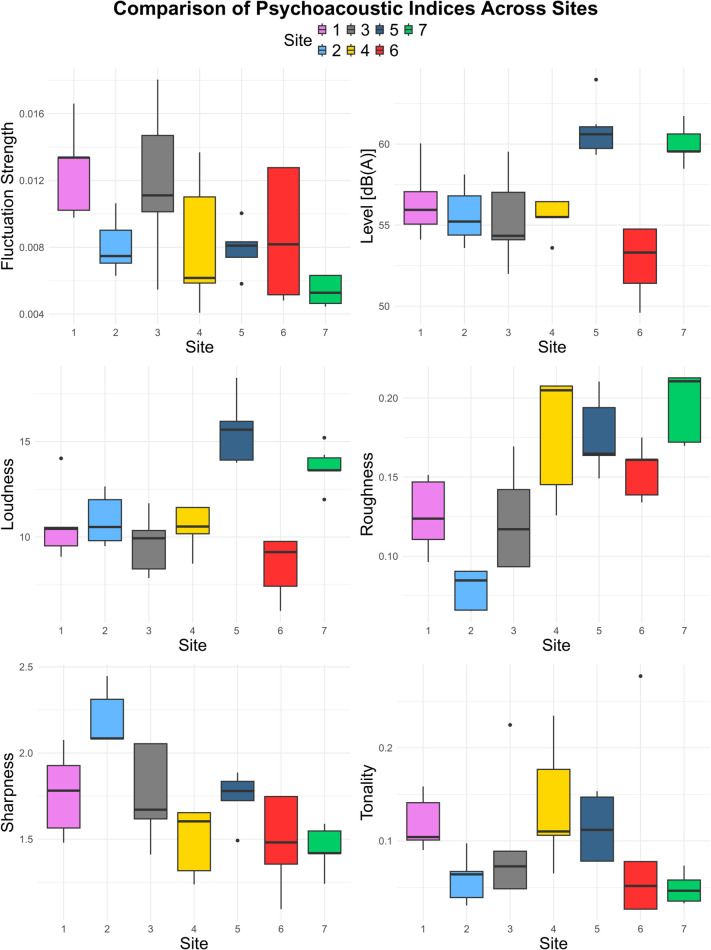
Boxplot of each psychoacoustic indices by site.

**Fig 3:**
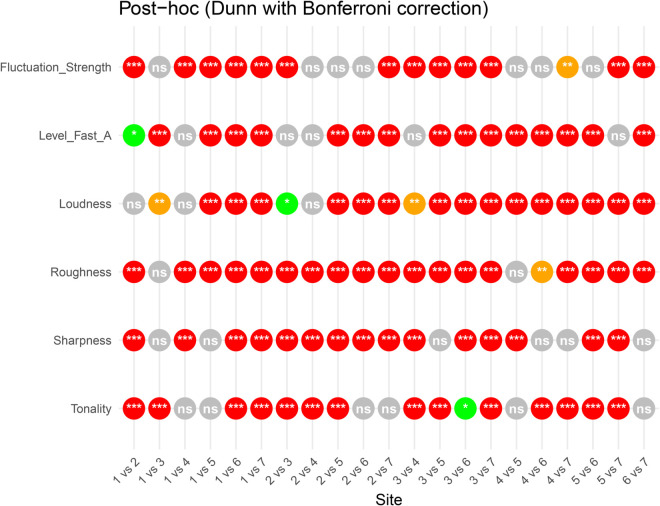
Pairwise test between Psychoacoustic indices and the different sites of the soundwalk (Dunn test with Bonferroni correction). Significance levels are color-coded as follows: red for *p* < 0.001 (***), orange for *p* < 0.01 (**), green for *p* < 0.05 (*), and gray for non-significant differences (ns).

The comparisons of the psychoacoustic indices (Table 1 and Fig 2) showed significant differences between sites (Kruskal-Wallis test *p* < 0.05 for all parameters). In detail, the post-hoc analysis (Fig 3) showed that Site 1 exhibits the highest fluctuation strength, indicating a stronger perception of amplitude fluctuations, while Site 2 stands out for the highest sharpness and low roughness values. Site 3 showed relatively low values across indices compared to all other sites, but exhibited more pronounced fluctuation values. Site 4 showed the highest and most variable tonality, high levels of roughness and low sharpness. Site 5, recorded the highest dBA level and loudness values, while Site 6 showed the lowest loudness and also sharpness indices. By last, Site 7 had the highest roughness levels, the lowest tonality and low fluctuation strength with minimal values with little variability.

**Table 1:** Mean and standard deviation of each psychoacoustic indices for each site.

Site	Level [dBA]	Loudness	Roughness	Fluctuation Strength	Sharpness	Tonality
1	56.51 ± 2.03	10.82 ± 1.81	0.13 ± 0.02	0.013 ± 0.003	1.78 ± 0.22	0.12 ± 0.03
2	55.29 ± 1.58	10.65 ± 1.19	0.08 ± 0.01	0.008 ± 0.001	2.20 ± 0.14	0.06 ± 0.02
3	55.44 ± 2.51	9.72 ± 1.38	0.13 ± 0.03	0.013 ± 0.004	1.71 ± 0.24	0.10 ± 0.07
4	55.45 ± 1.05	10.35 ± 1.04	0.18 ± 0.03	0.008 ± 0.003	1.47 ± 0.19	0.14 ± 0.06
5	60.63 ± 1.44	15.34 ± 1.49	0.17 ± 0.02	0.008 ± 0.001	1.73 ± 0.14	0.12 ± 0.03
6	52.66 ± 2.04	8.40 ± 1.48	0.15 ± 0.02	0.008 ± 0.003	1.45 ± 0.24	0.08 ± 0.08
7	59.80 ± 1.04	13.48 ± 1.02	0.19 ± 0.02	0.005 ± 0.001	1.44 ± 0.13	0.05 ± 0.01

**Fig 4:**
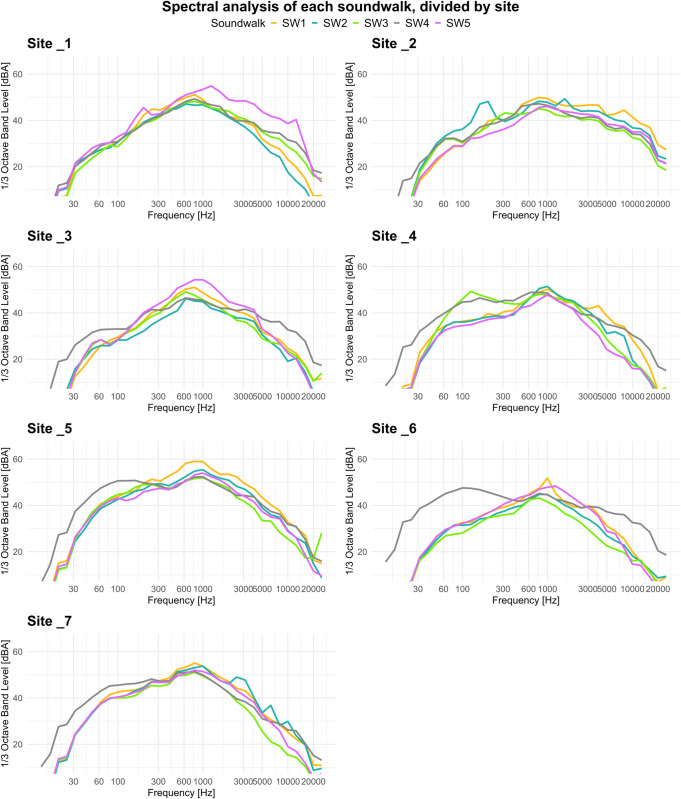
Spectral analysis of A-weighted spectral levels in the one-third octave bands frequency for each soundwalk and by site.

**Fig 5:**
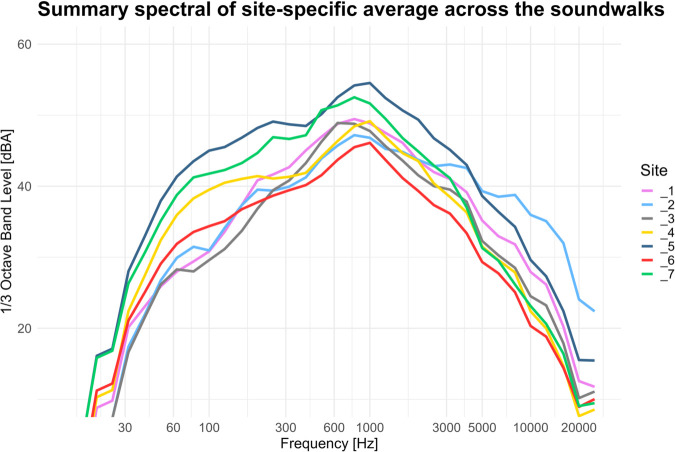
Mean one-third octave band spectra calculated for each site by averaging across all soundwalk sessions.

The spectral analysis revealed notable differences between the measurement sites (Fig 4). In particular, Soundwalk 4 exhibited higher sound pressure levels in the low-frequency range compared to the other sessions, at Sites 3, 4, 5, 6, and 7. Additionally, the high-frequency components at Site 2 appear more prominent than at the other sites. In the summary spectral chart grouped by site (Fig 5), Sites 5 and 7, corresponding to the intersection and Piazza della Scienza, exhibit higher intensity levels in the low-frequency range, with differences reaching up to 10 dB compared to Sites 1, 2, and 3. Site 4, located in the urban park, shows intermediate low-frequency levels between the two previously mentioned groups. In the mid-to-high frequency range, Site 2 (Piazzetta Difesa per le Donne) stands out with elevated intensity levels.

**Fig 6:**
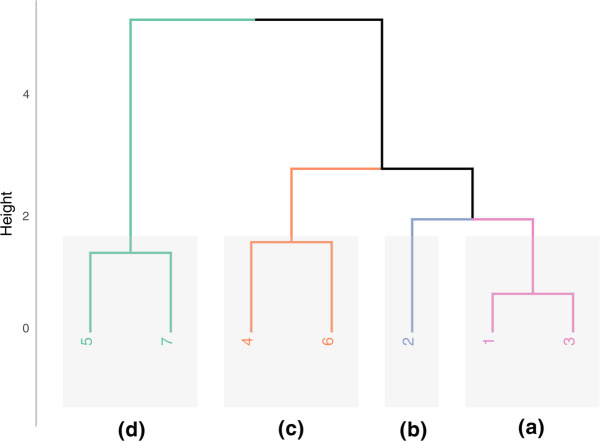
Dendrogram of cluster analysis between psychoacoustic measurements and sites. Each colour indicates a cluster (a–d).

The cluster analysis results are shown in a dendrogram, where similar sites are grouped into four clusters (Fig 6). The first includes Site 5 (Intersection of Viale Sarca and Via Luigi Emanueli) and Site 7 (Piazza della Scienza) while the second cluster isolated Site 2 (Piazzetta Difesa per le Donne). The third cluster grouped Sites 1 and 3 (Piazza dell’Ateneo Nuovo and Piazza della Trivulziana), and the last one grouped Sites 4 and 6 (Parco Sarca and Collina dei Ciliegi).

### Soundscape perceptions

**Fig 7:**
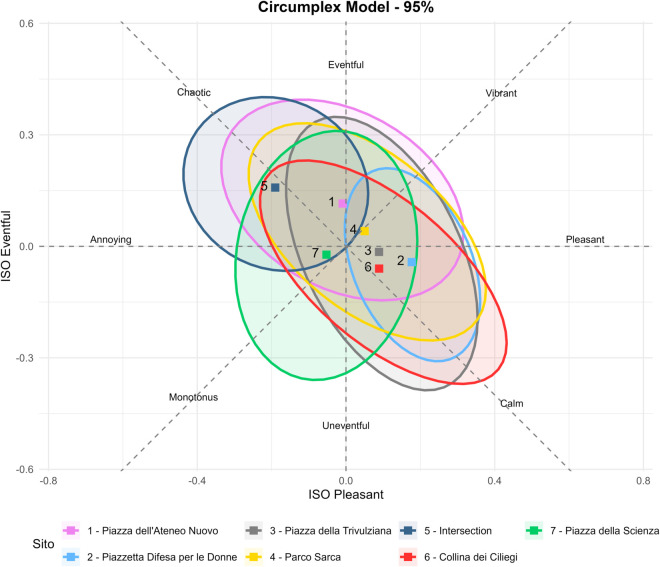
Circumplex Model of the sensations described by the participants in the seven sites. Ellipses represent data-driven 95% confidence regions for each site, computed from the empirical distribution of ISO Pleasant and ISO Eventful ratings (R, stat_ellipse, type = ‘t’).

Observing the two-dimensional model in Fig 7, Site 5 clearly emerged as the location perceived by participants as the most chaotic. Conversely, Site 2 was identified as the most pleasant, followed by Sites 3 and 6, with the latter also being frequently described as calm. Site 7 was commonly rated as monotonous and uneventful by a significant number of respondents. In contrast, Sites 1 and 4 were located in the quadrant associated with eventful and vibrant perceptions.

The correlation analysis conducted using Spearman’s rank correlation coefficient identified several significant associations (*p* < 0.01) between questionnaire responses, psychoacoustic indices, and A-weighted sound pressure levels (dB(A)), considering only correlations with |r|>0.4. The perceived loudness of the site, as reported on a 1-5 scale in participants’ questionnaire responses, showed significant positive correlations with A-weighted sound pressure levels (r = 0.514), loudness (r = 0.450), and tonality (r = 0.420). The rating of the participant answers regarding how unpleasantness they perceived each site was positively correlated with A-weighted sound pressure levels (r = 0.474) and loudness (r = 0.439). Regarding the ISO standardized scales, ISO Pleasantness scores were negatively correlated with A-weighted sound pressure levels (r=−0.517), loudness (r=−0.456), and roughness (r=−0.403).

**Fig 8:**
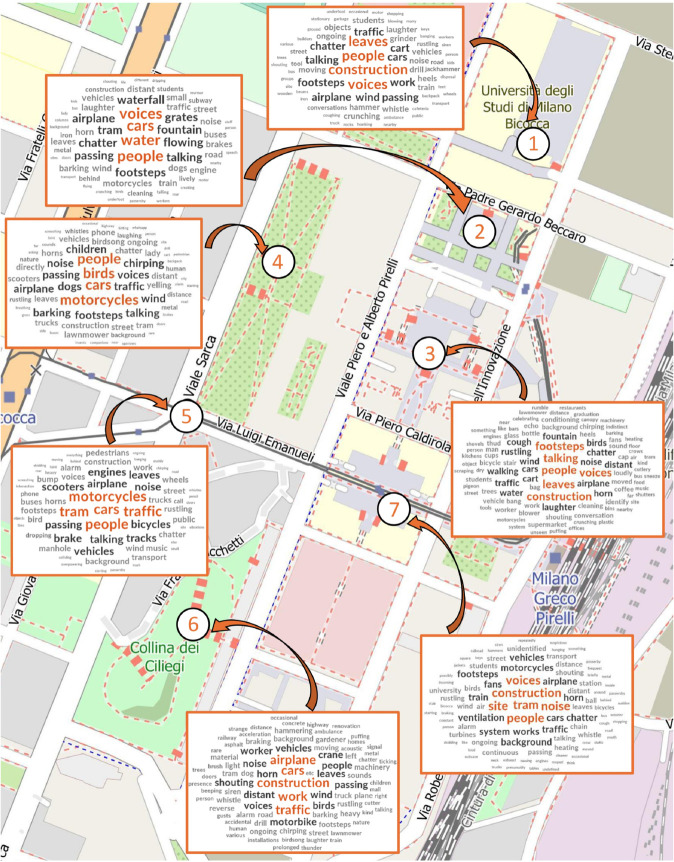
Map of the perceived sound sources per site (1-Piazza dell’Ateneo Nuovo, 2-Piazzetta Difesa per le Donne, 3-Piazza della Trivulziana, 4-Parco Sarca, 5-Intersection, 6-Collina dei Ciliegi, 7-Piazza della Scienza. Source: our elaboration of survey data. Basemap OpenStreetMap contributors (ODbL, https://www.openstreetmap.org/copyright). Map created with QGIS 3.38 Firenze.

Finally, ISO Eventfulness was positively correlated with tonality (r = 0.489). The average perception of the noisiness in the seven sites was slightly below moderate (2.85), with significant differences between the sites (X2 (24)= 297.98, *p* < 0.01). Site 5 was perceived as the loudest (80% of the responses were high or extremely noisy), while Site 2 (77% slightly or not noisy at all), 6 (62% slightly or not noisy at all) and 3 (53%) were considered as the least noisy. Perceived site noisiness was highly correlated with the perceived disturbance of the sound events (0.702, *p* < 0.01), with ISO eventful (0.625, *p* < 0.01) and negatively correlated with ISO pleasantness (–0.724, *p* < 0.01) and, to a lesser extent, to the appropriateness of the place to the context (–0.154, *p* < 0.01). The main perceived sound source vary across the sites (Fig 8): in Site 1 people’s voices, construction sites and leaves; in Site 2 peoples’ voices and footsteps, cars and fountain water; in Site 3 peoples’ voices and footsteps, construction sites and leaves; in Site 4 cars and motorcycles traffic, people and birds; in Site 5 motorcycles and cars traffic, tram and people’s voices; in Site 6 construction sites, cars traffic and airplanes; in Site 7 peoples’ voices, construction sites and ventilations system.

### Focus group

The main results of the five focus groups are presented according to three categories: squares (sites 1, 2, 3, 7), parks (sites 4, 6) and roads (site 7). In site 1, participants distinguished between sounds that were clearly audible, yet visually imperceptible, for example “the electric saw in construction sites” (29.10.2024-S1), and visually prominent sounds, yet not distinctly perceived at the acoustic level, such as “[... ] people’s footsteps, the rustling of leaves [... ]” (29.10.2024-S2). The artificial waterfall characterising site 2 was perceived as “an acoustic link between the urban and natural realms” with flowing water allowing students to “to calm down considerably” (29.10.2024-S3), except nearby the main road where traffic disturbs this sense of calm. Site 3 is characterized by human activity, such as voices from cafés or the clinking of cups and saucers, perceived as less disturbing and acquiring meaning for individuals.

Site 7 stood out for being acoustically dominated by the continuous and pervasive “white noise” of ventilation and air-conditioning systems, compounded by the square’s architectural configuration. “The structure is basically a square and, if you stand in the middle, everything just echoes back at you” (21.10.2024-S2). Site 4 was reported as a space potentially suited for studying and relaxation, yet compromised by its proximity to the road, which hindered its usability. As one participant noted: “in the end you hear the same sounds as at the intersection. Sure, you hear the birds, but also the same traffic noise. It almost bothered me more. You think, damn, I’m in a park, but I’m basically on the street” (21.10.2024-S3).

In site 6, participants reported a similar perception, marked by the hill’s elevated position and incongruity with the visual context. The former allows for a clearer perception of the contrast between the more muffled sounds of the upper areas and the louder, more chaotic ones coming from the surroundings. The latter points to a perceived dissonance between visual and acoustic stimuli, namely visual elements of the park and acoustic disturbance similar to a road intersection.

Site 5 is described as a familiar element that students “don’t even notice anymore. It’s just there. (...) I’m used to it” (28.11.2024-S3) as well as an accumulation of acoustically dissonant factors. “There are a lot of stop signs, which sometimes aren’t respected, honking, people accelerating, and various other noises... ” (21.10.2024-S6). One participant emphasized the varied impact of different components of traffic noise: “The engine is annoying, but then, when you shift gears, it starts to make less noise; whereas the tires, if you’re going fast, they’re just annoying” (21.10.2024-S8). This negative perception also influences how nearby commercial spaces are used, for example avoiding eating at a local pizzeria with outdoor seating.

## Discussion

The integration of acoustic, psychoacoustic and perceptual data provided a comprehensive characterization of the soundscapes encountered during the soundwalk conducted in the Bicocca university district. Overall, each site had unique characteristics, but the combination of the main features grouped the green areas together as well as the most urbanized and disturbed sites (Table 2).

**Table 2:** Summary of the main data from the results divided by site. Full data is avaiable in SI3.

Site	Cluster	Level [dBA]	Perceived - How noisy is the site?	Circumplex Model	Sound Sources (Perceived)	Psychoacoustic indices
1	a	56.51	3.09	Eventful	Construction, Voices, People, Leaves.	High Fluctuation Strength
2	b	55.29	2.20	Most Pleasant	Voices, Cars, Water, People.	High Sharpness Low Roughness
3	a	55.44	2.43	Pleasant	Footsteps, Talking, People, Voices, Leaves, Construction.	All indicators low
4	c	55.45	3.17	Vibrant - Eventful	People, Birds, Cars, Motorcycles.	High Tonality High Roughness
5	d	60.63	4.09	Most Chaotic	Motorcycles, Tram, Cars, Traffic.	High Loudness High Roughness
6	c	52.66	2.31	Pleasant - Calm	Airplane, Cars, Traffic, Work, Construction.	Low Loudness Low Sharpness
7	d	59.8	3.06	Most Monotonous	Voices, Construction, Site, Tram, Noise, Ventilation.	High Loudness High Roughness Low Tonality

More specifically, the soundscape at Site 1 (Piazza dell’Ateneo Nuovo) was strongly influenced by nearby construction noise. Indeed, this site exhibited the highest level of fluctuation strength, which can be associated with slow amplitude modulations, typically perceived as pulsating or trembling sounds [22]. This suggests a marked perception of amplitude variations, likely linked to the intermittent character of construction site sounds, such as those produced by a concrete pump [35]. According to the circumplex model of soundscape perception, this site was rated as the most eventful among all locations. Natural sounds such as human voices and rustling of leaves can be masked by mechanical sources like jackhammers and concrete pumps, leading to a perceptual mismatch between the calm visual setting and the intrusive auditory experience [36].

Despite exhibiting a relatively low sound pressure level, ranking as the second quietest site after Site 6, Site 2 (Piazzetta Difesa per le Donne) was perceived by participants as the least noisy and most pleasant, according to the circumplex model. Boxplot analysis revealed the highest sharpness levels, indicating a soundscape dominated by high-frequency components, and the lowest roughness values, indicating a lack of fast amplitude modulations. These acoustic and perceptual characteristics made Site 2 stand out as a distinct cluster in the multivariate analysis.

Spectral analysis further showed high-frequency content probably associated with the artificial waterfall. Previous studies have demonstrated that water features in urban environments can enhance perceived acoustic quality by effectively masking road traffic noise [37,38]. In addition to water features, natural elements in green spaces, such as those observed in Sites 2, 3, 4, and 6, have been associated with positive effects on human health, well-being, and quality of life across physical, psychological, and social domains [37,38]. In both the survey and the focus group, participants consistently identified the artificial waterfall as a soothing auditory element, often evoking associations with natural environments. Accordingly, studies have found that the presence of water not only makes noise seem less intrusive but also improves how people perceive the overall sound quality—even in areas with heavy traffic [39,40]. Furthermore, the spatial configuration of the square was perceived as functioning effectively as an acoustic buffer, mitigating the impact of nearby traffic, tram noise, and construction activity [41].

Psychoacoustic indicators measured at Site 3 (Piazza della Trivulziana) were generally lower than those observed at the other sites. This suggests that the area is comparatively quiet, likely due to its configuration as a sunken courtyard, which effectively reduces the intrusion of external noise sources. According to [42], sunken courtyards can significantly reduce the perception of traffic noise and are often experienced as acoustically comfortable environments. In the Circumplex model, the centroid of Site 3 is positioned near the center of the graph, close to Site 6. This placement suggests a balanced perceptual profile, likely reflecting the site’s calm atmosphere and the contribution of vegetation and natural acoustic elements. This interpretation is consistent with [43], who found that vegetation and spatial layout in urban green areas significantly influence the perception of calm and acoustic harmony. The most prominent sound sources at this site included human voices, rustling leaves and footsteps, all contributing to a soundscape that was both serene and socially engaging. Overall, the soundscape was deemed particularly conducive to fostering social interaction and conviviality.

The soundscape of Site 4 (Parco Sarca) was heavily influenced by the constant traffic noise from Viale Sarca, which runs parallel to the entire length of the park. According to [44], the psychoacoustic indices of roughness are elevated, likely due to the high speed of vehicular traffic along the roadway. The tonality index was also relatively high at this site, indicating the presence of prominent tonal components within the sound environment. These components may stem from localized mechanical or infrastructural sources (e.g., HVAC systems, electronic beeps, or steady tonal emissions) and align with the literature in previous studies [45]. In the cluster analysis, Sites 4 and 6, both classified as urban parks, are grouped within the same cluster. However, in the Circumplex model, Site 4 stands apart from Sites 2, 3, and 6, tending to be perceived as more eventful and vibrant, likely due to its variety of sounds. At this site, the most prominently perceived sound sources, in addition to birdsong [46], included human voices, along with traffic noise from cars and motorcycles. During the focus group discussions, participants highlighted that the parks, and in particular Site 4, did not meet their acoustic expectations, as they perceived several artificial noises in green spaces that, in their view, should be calmer and characterized by natural sounds. At Site 5 (intersection of Viale Sarca and Via Luigi Emanueli), the highest sound pressure level was observed, along with the highest loudness. Roughness was also notably high. According to [44], these acoustic characteristics are likely attributable to the combined effect of vehicular traffic and tram line operations in the vicinity. In the circumplex model, the perceptual centroid of Site 5 corresponds to a highly chaotic auditory environment, dominated by noise from cars, trams, and motorcycles. Cluster analysis groups Site 5 with Site 7 (Piazza della Scienza), possibly due to similar acoustic conditions. Piazza della Scienza is likewise exposed to significant traffic noise from surrounding roads, and to tram noise due to a tram stop located in the center of the square. During the focus group, participants agreed that Site 5 was the most bothersome location of the walk and not suitable for social activities. Moreover, some participants clearly distinguished between different traffic noise sources: the sound of engines as cars restarted at the traffic light, the engine noise of motorcycles and the noise of tires from circulating vehicles. In the discussion, the speed of the cars and the consequent noise of the cars was considered as the main cause of noise.

Site 6 (Collina dei Ciliegi) showed the lowest values of sound pressure level, loudness, and sharpness. Although the site is located within a green area, typically associated with lower acoustic intensity, the predominant perceived sounds were traffic noise and construction activity. The site’s elevation likely contributes to the increased audibility of distant sound sources [47]. Cluster analysis appears to reflect this duality by grouping Site 4 (Parco Sarca) with Site 6. The spectral low-frequency values observed particularly in Soundwalk 4 graphs possibly related to the elevated wind, despite wind speed measurements remaining within the regulatory limits thresholds defined by D.M. 16/03/98 [48] and UNI EN ISO 1996-1:2017 [49]. In the Circumplex model, the centroid of Site 6 was located close to Site 3, but it was perceived as more calm. In general, the soundscape of the Collina dei Ciliegi was considered slightly disappointing, for reasons similar to those already discussed for Site 4. During focus group discussion, participants debated the park’s soundscape: some found comfort in the muffled noises of the distant construction sites, describing a sense of being in a “bubble” that protected them from the chaotic city, while others felt “betrayed” by the lack of quiet in a space that, in their view, should have offered a more natural experience.

The final stop of the soundwalk was Site 7 (Piazza della Scienza), which was strongly impacted by noise emissions from technical installations (RTU – roof top unit) located on the surrounding university buildings. This site has been previously discussed in greater detail in a previous study [50]. The high roughness and low tonality suggest a scarcity of distinct tonal components relative to the background noise generated by RTU. The sound pressure level and loudness were also high at this site, likely contributing to the grouping of Site 7 with Site 5 (intersection) in the cluster analysis. In the circumplex model, this site was perceived as the most monotonous, likely due to the limited variety of auditory stimuli. During the focus group discussions, the constant noise produced by the ventilation systems was regarded as a factor that detracts from conviviality and impairs the quality of social interactions in the space. However, in one group discussion, divergent perspectives emerged: while some participants described the ventilation noise as annoying and oppressive, lingering even after leaving the area (‘in my ears I hear this... this annoyance’, 29.10.2024-S3), others acknowledged its potential mitigating effect on the acoustic variability of the surrounding environment, perceiving it as less disturbing than sudden noises such as engines or braking from road traffic.

Overall, the focus group discussions underscored the importance of participants’ expectations and familiarity with the location in shaping their soundscape evaluations [51,52]. Regarding expectations, parks represented a notable case. Being consistently associated with tranquility and natural sounds, participants often expressed surprise, or even irritation, when the actual soundscape of such green spaces diverged from these expectations. This aligns with previous research highlighting that the perception of park soundscapes is particularly influenced by personal factors including the degree to which expectations are met [53]. Survey data further supported these findings, revealing a heightened sensitivity to noise in park environments. For instance, participants reported a higher perceived noise level at Collina dei Ciliegi compared to Piazzetta Difesa delle Donne, despite the former exhibiting a lower average measured sound level (52.7 dBA vs. 55.3 dBA). A similar pattern was observed at Parco Sarca, which was perceived as noisier than all other locations except for Site 5 (the road intersection), even though its average recorded sound level was comparable to that of Piazza della Trivulziana, lower than Piazza dell’Ateneo Nuovo, and substantially lower than Piazza della Scienza.

In contrast, noise in urban squares was generally well tolerated, provided it stemmed from human voices and social activities, suggesting positive perceptions of sounds when these are associated with expected social uses of public spaces. In a similar vein, sounds from construction sites and ventilation systems became significantly more disturbing when they overpowered conversational sounds and impeded social interaction.

Among students, a key factor in evaluating the university district’s soundscape was familiarity with dense urban contexts, in particular Milan. Participants’ geographical origin prominently emerged during the focus groups, pointing to a dichotomy between rural and urban backgrounds which recalled distinct perceptions of sonic environments. In particular, the contrast was between the quietness of rural and peripheral contexts and the constant noise experienced in densely populated cities. While students from small villages in the South of Italy, “where everything is perpetually quiet, [found that] this kind of constant noise has never been part of [their] life” (28.10.2024-S3), those from suburban locations perceived the examined soundscape, particularly at park Collina dei Ciliegi, more similar to their hometown. These participants envisioned Milan as an “amplified version” of the suburban soundscape they are used to. Speaking of a main road, viale Fulvio Testi, one underlined that “while in my hometown urban traffic stops at 3 am, here it never stops” (29.10.2024-S3). Familiarity with space in the perceptions of soundscapes is further entangled with personal memories, e.g. experience of pandemic times, and adaptation processes, which span both spatial and temporal dimensions in individual biographies. For instance, one interviewee from Southern Italy highlighted the initial difficulty in adapting to the Milan urban soundscape, describing it as a drastic change. However, over time, this sonic frenzy became familiar: “At first, I wasn’t used to it because, after all, it’s a sharp, radical change. But... it’s been almost three years now, and you get used to it. I mean, by now I don’t even notice it anymore.” (28.10.2024-S3).

## Conclusions

This paper investigated the soundscape of a university district in Milan, Italy, drawing on multiple research methods, including quantitative analysis on acoustic and psychoacoustic indicators, survey data, and qualitative focus groups. It explored the spatio-temporal interactions between sound environments, places, daily practices and subjective experiences. The different research methods adopted in this study allowed us to grasp multiple dimensions of the soundscapes and the multifaceted experiences of their users, mainly students. Considering each site of the soundwalk, these methods pointed to overall similar results, suggesting that the combination of quantitative and qualitative noise assessment can provide deep and reliable information to guide policymaking in this field. Across selected sites, the analyses intuitively revealed some remarkable differences.

Participants showed a marked preference for locations incorporating natural elements, such as the urban parks at Sites 4 (Parco Sarca) and 6 (Collina dei Ciliegi), as well as the artificial waterfalls at Site 2. However, in the urban parks, traffic and construction noise were reported as detracting factors, compromising the otherwise restorative and pleasant qualities of these environments. Site 2 (Piazzetta Difesa per le Donne) emerged as the most appreciated and perceived as the most relaxing location, likely due to the continuous presence of the waterfalls sound and its recessed positioning, both of which effectively masked the intrusive urban noise.

This study confirmed the hypothesis that human sound perception is influenced by contextual factors and cannot be solely explained by objective parameters such as sound pressure level. It also highlighted the importance of understanding how people make sense of sounds, how they attribute particular meanings and how such processes are tied with prior sound experiences and biographical memories. The different sounds perceived across the sites, e.g. human voices and footsteps, construction sites, leaves and motor traffic, play a key role in shaping a different use of public space, either facilitating aggregation, enabling relaxation or conversely hampering social interaction.

Improving the perceptual quality of the soundscape requires more than a mere reduction of noise levels, it necessitates a qualitative refinement of the acoustic environment. The implications of these findings are therefore pivotal for designing public policies in the fields of urban planning as well as its incorporation within broader regeneration strategies [54], particularly in proximity to university campuses.

This study contributed to sound research by proposing a new approach to study soundscapes that consider students as the main users and university districts as the main auditory environment. Following [55], this paper reflected on some aspects of the individual characteristics of users, e.g. geographical background, their uses and familiarity with the space, as well as the contrast between perceived and expected auditory experience. In terms of planning strategies, university districts as an auditory environment shall account for students’ need for quiet areas conducive to study. Thus, the enhancement of outdoor sound environments emerges as a critical factor influencing academic performance and socialisation during university years.

## Supporting information

S1 File Soundwalk questionnaire items. This PDF file reports the full list of questionnaire items used during the soundwalks.
(PDF)

S2 File Focus Group Questions. This PDF file reports the questions used during the focus groups conducted at the end of the soundwalks.
(PDF)

S3 Table Complete dataset of responses and analyses performed. For each soundwalk and for each specific site, the table reports the corresponding data: soundwalk questionnaire responses and the related ISO Pleasant and Eventful values, psychoacoustic indices, and meteorological data.
(XLSX)
