# Supplementary material for: Walk and listen: A multidimensional study on the soundscape of a University District
Source: PLoS One. 2026 Feb 20;21(2):e0343065. doi: 10.1371/journal.pone.0343065 (PMC12923137; doi:10.1371/journal.pone.0343065)
Supplement: S1 File — This PDF file reports the full list of questionnaire items used during the soundwalks. (PDF) [file pone.0343065.s001.pdf]

Soundwalk questionnaire items

1. Gender
2. Age
3. Occupation
4. How noisy is this place?
5. Regarding the sound environment around you, to what extent do you agree or disagree with each of the following 8 attributes:
   5.1. Pleasant, comfortable
   5.2. Chaotic, confusing
   5.3. Eventful, stimulating
   5.4. Uneventful, stationary
   5.5. Calm, peaceful
   5.6. Annoying, irritating
   5.7. Vibrant, varied
   5.8. Monotonous, boring
6. List the sound sources you noticed in this place (up to a maximum of 8), from the most salient to the least salient.
7. Did you perceive any evident sound events during listening, that is, sudden and brief noises (e.g., horns, bangs, screams, screeches, puffs, etc.)?
8. How disturbing do you find the sound events you heard?
9. How unpleasant is this place?
10. How appropriate is this place to its context?
11. Would you visit this place again?
12. What are you thinking? Describe your thoughts and feelings after listening to the sound environment.
