# Supplementary material for: Walk and listen: A multidimensional study on the soundscape of a University District
Source: PLoS One. 2026 Feb 20;21(2):e0343065. doi: 10.1371/journal.pone.0343065 (PMC12923137; doi:10.1371/journal.pone.0343065)
Supplement: S2 File — This PDF file reports the questions used during the focus groups conducted at the end of the soundwalks. (PDF) [file pone.0343065.s002.pdf]

Focus Group Questions

1. Did you notice anything different when you focused on sounds rather than sights? What? Did your surroundings seem different? How?
2. What memories did the sounds you heard bring back? Did any of them evoke experiences, places or feelings from your past? Can you describe them?
3. How did the different sounds you heard during the walk affect you? Do they remind you of any moments in your daily life and places in this neighbourhood?
4. Were there any sounds that you found disturbing or out of place? How do you think these sounds affect the way people live in the neighbourhood, e.g. quality of life or social life?
5. Do you think that certain groups may benefit/be penalised more by the sounds you hear (or heard) every day? Can you tell us about it?
